# Genetic Dissection of Frost Tolerance in Winter Durum Wheat: Three Validated KASP Markers for Marker-Assisted Selection

**DOI:** 10.3390/plants15010019

**Published:** 2025-12-20

**Authors:** Mikhail Divashuk, Aleksey Ermolaev, Viktoria Voronezhskaya, Aleksey Yanovsky, Varvara Korobkova, Ludmila Bespalova, Alexandra Mudrova, Anastasiya Voropaeva, Anastasia Lappo, Stepan Toshchakov, Mariia Samarina, Anastasia Krylova, Gennady Karlov

**Affiliations:** 1 All-Russian Research Institute of Agricultural Biotechnology, Moscow 127550, Russia; alerm@al-erm.ru (A.E.); voronezhskaya.vs@gmail.com (V.V.); samarina.homa@yandex.ru (M.S.);; 2 National Research Center “Kurchatov Institute”, Moscow 123182, Russia; toschakov_sv@rrcki.ru (S.T.);; 3 School of Biological and Medical Physics, Moscow Institute of Physics and Technology, Moscow 141701, Russia; 4 P.P. Lukyanenko National Grain Center, Krasnodar 350012, Russia

**Keywords:** *Triticum turgidum*, GWAS, KASP, frost-resistant, durum wheat, LRT

## Abstract

Winter durum wheat combines the benefits of autumn sowing with high grain quality but remains poorly adapted to temperate regions due to low frost tolerance. To elucidate the genetic basis of winter hardiness and support breeding for improved cold adaptation, a segregating multi-family F_2_ panel (n = 270) was developed from crosses among frost-tolerant and frost-susceptible lines. GWAS identified four loci significantly associated with winter survival on chromosomes 1B, 5A, 5B, and 7B, collectively explaining 7.6–21.5% of phenotypic variance. These loci jointly improved model performance (ΔMcFadden R^2^ = 0.230, *p*-value = 4.76 × 10^−17^) without evidence of epistasis, indicating additive inheritance. Predicted survival increased nearly linearly with the number of favorable alleles, highlighting the potential for pyramiding through marker-assisted or genomic selection. Three significant SNPs were converted to KASP assays, providing validated molecular tools for breeding applications. Overall, the study broadens understanding of frost-tolerance genetics in winter durum wheat beyond canonical *Fr* regions and delivers practical markers for improving winter hardiness in breeding programs targeting continental climates.

## 1. Introduction

Durum wheat (*Triticum turgidum* L. subsp. *durum* (Desf.) Husn.) is one of the world’s most important cereal crops and is highly valued by the food industry for pasta and semolina production [1]. Despite the economic importance of durum wheat, most modern cultivars belong to the spring growth habit, whereas the development of winter durum wheat is relatively recent. The breeding of winter-hardy durum forms began only in the second half of the 20th century and remains limited compared with the long-established improvement of winter bread wheat (*Triticum aestivum*) [2,3].

Our previous analyses of winter durum wheat have identified substantial allelic diversity at *Glu-1* loci affecting grain quality, indicating that this germplasm holds considerable potential for quality improvement [4]. However, the full realization of this potential, coupled with consistent yield performance, is significantly hindered by sensitivity to low temperatures. Because of a shorter breeding history, winter durum germplasm still exhibits considerable variability in cold tolerance, and the physiological and genetic mechanisms underlying its winter survival are not yet well characterized. For example, spring frosts are well known for causing sterility in spikes and damaging reproductive organs, which directly reduces grain growth process [5].

The creation of frost-resistant winter durum varieties is therefore of high strategic importance: winter forms can exploit the full vegetation period, achieve higher yield potential, and contribute to the diversification of durum wheat cultivation in colder continental regions [6,7,8]. Owing to generally lower frost tolerance compared with bread wheat, durum wheat is considered more sensitive to cold stress [8,9]. Therefore, improving frost tolerance in durum wheat is a critical breeding objective to ensure stable productivity and to expand cultivation into colder environments.

The ability of wheat plants to withstand subzero temperatures is governed by cold acclimation—a process whereby gradual cooling triggers metabolic reprogramming and accumulation of protective compounds, thereby enhancing subsequent frost tolerance [10]. Frost tolerance is a quantitative, polygenic trait controlled by numerous loci whose effects interact with the environment. Major loci of frost resistance reside on chromosome 5. In particular, Frost Resistance-1 (*Fr-1*) and Frost Resistance-2 (*Fr-2*) on the long arm of 5A are key contributors: *Fr-1* is linked to growth habit (winter vs. spring) by vernalization genes such as *Vrn-A1*, whereas *Fr-2* encompasses the CBF (C-repeat Binding Factor genes) gene cluster and exerts the strongest influence on frost and winter survival [11].

Consistent with this, Gupta et al. (2020) demonstrated that although *Vrn-A1* and photoperiod genes *Ppd-A1*/*Ppd-B1* are major determinants of flowering in durum wheat, they explain only part of the phenotypic variation, implying the existence of additional polygenic regulators within the vernalization network that may also influence cold adaptation [12]. Indeed, the *Fr* loci contribution to frost tolerance is strongly influenced by developmental stage and environmental conditions, thereby contributing to the developmental and environmental dependence of *Fr-1* loci effects. Ferrante et al. (2021) demonstrated that in winter wheat, the *Fr-1* locus confers high frost tolerance during the vegetative phase (below −12 °C), but its regulatory effectiveness declines after vernalization as plants enter the reproductive stage [13]. The *Fr-2* region, in turn, exhibits strong linkage disequilibrium and limited allelic diversity, with only two major haplotypes identified in modern winter wheat [11,14]. The superior haplotype provides approximately 15% higher winter survival compared to the alternative one, suggesting that most of the favorable variation within this cluster has already been exploited. As a result, further improvement in frost tolerance through *Fr* loci alone may reach a plateau, emphasizing the importance of identifying additional QTL operating independently of the *Fr*-mediated pathway to achieve complementary genetic gains and enhance the resilience of durum wheat to frost stress.

Genome-wide association studies (GWAS) are widely used to dissect the genetic architecture of complex traits in crops, and frost tolerance is no exception. Despite considerable progress in bread wheat, comparable data for durum wheat remain scarce. The objective of this study was to identify genomic loci associated with frost tolerance in winter durum wheat and to develop molecular markers for selecting highly tolerant genotypes in breeding programs, including loci beyond the well-characterized *Fr*-regions to broaden the genetic basis of frost tolerance. Following the methodological framework established in our previous study on spring durum wheat [15], we conducted a GWAS to assess the survival of winter durum wheat under frost conditions. The analysis revealed a novel genomic loci associated with frost resistance. Subsequently, Kompetitive Allele-Specific PCR (KASP) markers were developed for the identified allelic variant, and their efficiency for marker-assisted selection was validated.

## 2. Results

### 2.1. Multiple Significant SNP Associations with Frost Tolerance

**Figure 1:**
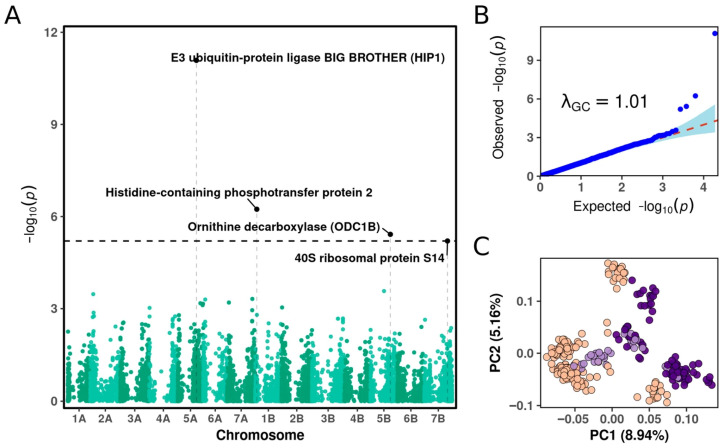
Genome-wide association analysis of frost tolerance in winter durum wheat. (**A**) Manhattan plot showing SNP associations identified using the BLINK algorithm. The horizontal dashed line indicates the FDR threshold of 10%, and annotated loci correspond to candidate genes with significant associations. Vertical dashed lines mark the genomic positions of SNPs significantly associated with frost tolerance. (**B**) Quantile–quantile (Q-Q) plot of expected versus observed –log_10_(*p*-values). The red dashed line represents the null expectation under no association, and the shaded area indicates the 95% confidence interval. The genomic inflation factor (λ_GC_ = 1.01) indicates minimal inflation due to population structure. (**C**) Population structure PCA plot of the durum wheat panel used for GWAS. Each data point represents an individual F_2_ genotype: progeny derived from cultivar Tsel (peach), progeny derived from Senora (violet), and admixed individuals carrying alleles from both parental lines (lavender).

The BLINK algorithm with an FDR threshold of 10% identified four SNPs significantly associated with frost tolerance in winter durum wheat. Significant associations were located on chromosomes 1B, 5A, 5B, and 7B (Figure 1A, Table 1). The percentage of variance explained (PVE) by these loci ranged from 7.6% to 21.5%, indicating a substantial contribution to winter survival. The quantile–quantile (Q-Q) plot showed a close fit between observed and expected *p*-values (λ_GC_ = 1.01), suggesting the absence of *p*-value inflation and confirming the robustness of the association model (Figure 1B). The first two principal components explained approximately 8.9% and 5.1% of the total genetic variance, respectively (Figure 1C). Principal component analysis (PCA) revealed clear genetic differentiation among individuals, based on a polymorphic panel of F_2_ progeny derived from the cultivars Tsel, Senora, and from both parental lines. Plant individuals with mixed ancestry from Tsel and Senora were positioned between the two main clusters, reflecting their hybrid genetic composition.

The identified SNPs, particularly those on chromosomes 5A and 7B, represent promising targets for marker-assisted selection aimed at improving winter hardiness. The four SNP loci considered in this study are provided in Supplementary Data S1.

### 2.2. KASP Validation Analyses

To confirm the identified associations, specific KASP markers were designed for four significant SNPs detected in the GWAS (Table 2). Three of the four KASP assays successfully amplified the target regions and clearly discriminated the genotypes, producing reproducible allele clusters with consistent amplification and accurate genotyping. One of the detected SNPs was located within a microsatellite repeat region, which made it impossible to design a KASP marker for this SNP.

**Figure 2:**
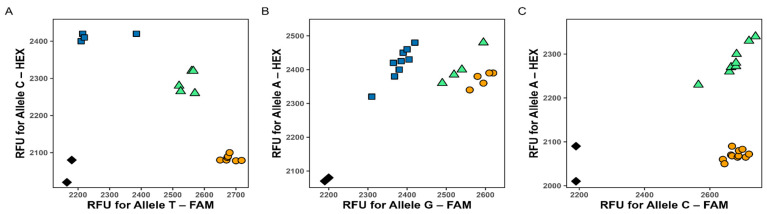
Results of each KASP markers visualization for: marker 1B_41099587 (**A**), marker 5B_517276534 (**B**), and marker 7B_598228866 (**C**). Each data point represents a genotype call: homozygous for the FAM-labeled allele (orange), homozygous for the HEX-labeled allele (blue), heterozygous (green), and no template controls (NTC, black diamonds). RFU stands for relative fluorescence units.

As shown in Figure 2, all three KASP assays demonstrated clear allelic discrimination and reproducible genotype clustering.

The marker 1B_41099587 (Figure 2A) showed well-defined clusters, indicating robust amplification and allele specificity. The marker 5B_517276534 (Figure 2B) also produced reliable results, although the HEX fluorescence signal was comparatively weaker, suggesting a minor imbalance in primer competition. The marker 7B_598228866 (Figure 2C) yielded two distinct genotype clusters corresponding to heterozygous and homozygous FAM-labeled individuals, indicating that the population structure comprised only these two genotype classes. Both clusters were clearly separated, confirming accurate genotype assignment.

Although KASP conversion failed for SNP 5A_487200180 due to its tandem repeat context, this locus was retained in post hoc analyses using its original GBS-derived genotypes, given its substantial effect size (PVE = 21.5%). The three successfully converted KASP markers (1B, 5B, 7B) provide validated tools for MAS, while the 5A locus requires alternative genotyping platforms (e.g., TaqMan, HRM, Sanger sequencing) for practical deployment.

### 2.3. Subsequent Validation Analyses

Post hoc logistic regression analyses were performed to evaluate the contribution of individual SNPs to survival probability (Table 3). A joint likelihood-ratio test (LRT) comparing the model of four SNPs with the null model confirmed a highly significant improvement in model fit (LRT = 82.7, df = 4, *p* = 4.76 × 10^−17^) with an overall ΔMcFadden R^2^ = 0.230.

Type II LRTs (drop-one tests) revealed that all four loci independently contributed to survival (Table 4). The strongest individual effects were detected for 5A_487200180, 5B_517276534 and 7B_598228866, while 1B_41099587 also showed a moderate but significant effect.

On the logit scale, the allelic effects (β) indicated that minor alleles at loci 5A_487200180, 7B_598228866 and 1B_41099587 were associated with increased survival probability, whereas the minor allele at 5B_517276534 was associated with decreased survival.

To explore potential epistatic interactions, we employed two complementary modeling strategies. First, we tested a comprehensive “Additive + All 2-way Interactions” logistic regression model, which incorporated pairwise interaction terms between all significant SNPs. This global approach allowed us to screen for non-additive effects across the full set of loci simultaneously. Pairwise interaction testing did not detect significant epistasis among the four loci (df = 6, *p* = 0.676; ΔR^2^ = 0.011), suggesting an additive mode of SNP contribution (Table 5).

To further assess the potential contribution of SNP 7B_598228866, located near a ribosomal protein S14 gene cluster, we constructed targeted interaction models of the form Additive + 7B_598228866 × SNP_i. This test was motivated by the hypothesis that translational regulation and membrane signaling pathways may jointly modulate cold stress adaptation [16]. The interaction between SNP 7B_598228866 and SNP 5B_517276534 displayed a *p*-value of 0.120, which was not statistically significant. However, the coefficient (β = −0.83) was substantial, suggesting a potential effect that was not captured by the significance threshold due to the limited sample size (Table 6).

This trend warrants further investigation. Given the limited sample size in the current model, it is plausible that the statistical power was insufficient to detect interaction effects robustly. Such efforts could refine our understanding of multigenic regulation in cold stress response (see Section 3).

Model comparisons of inheritance patterns supported additive or dominant effects for all four loci, with similar McFadden R^2^ values between models (Supplementary Table S1). Genotypic (2 df) models provided only marginal improvements, indicating no major deviation from additivity.

### 2.4. Predicted Probability of Survival Depending on Minor Allele Dosage

The predicted probability of survival was estimated using logistic regression models fitted separately for each significant SNP. This pattern is exactly what an additive model without epistasis would generate and aligns with our LRT outcome.

**Figure 3:**
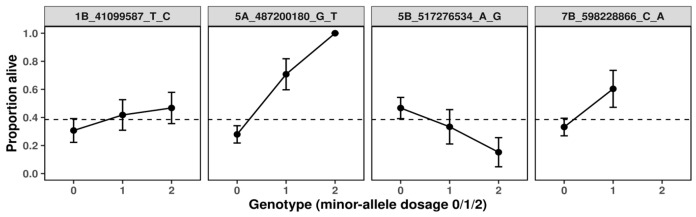
Predicted survival probabilities by genotype for frost tolerance-associated SNPs in winter durum wheat.

Distinct genotype–response patterns were observed across the four loci (Figure 3). At 1B_41099587, the probability of survival increased modestly with the number of minor alleles, suggesting a weak additive contribution to cold tolerance. Although the effect was consistent, its magnitude indicates that this locus likely plays a secondary, modulatory role within the broader genetic network. At 5B_517276534, an inverse trend was detected: individuals carrying one or two copies of the minor G allele exhibited a progressive decline in survival probability, indicating a detrimental additive effect. In sharp contrast, 5A_487200180 displayed a strikingly strong positive effect. The presence of the T allele markedly increased predicted survival, with heterozygotes showing substantially higher cold tolerance than non-carriers and homozygous T individuals reaching near-maximal predicted survival probabilities (>0.9). The steep allele–response curve and high model fit (McFadden’s R^2^ = 0.11) point to this variant as a major determinant of frost tolerance. Finally, 7B_598228866 exhibited a pattern similar in direction to 5A, with heterozygotes achieving the highest predicted survival among all loci examined. Although the homozygous-minor genotype was absent in this cohort, the magnitude of the heterozygous advantage implies an over dominant or regulatory effect potentially enhancing stress adaptation.

Generally, four favorable alleles associated with enhanced frost tolerance were identified in winter durum wheat: A at 7B_598228866 (β = 1.634), A at 5B_517276534 (β = −0.861), C at 1B_41099587 (β = 0.572), and T at 5A_487200180 (β = 2.094).

**Figure 4:**
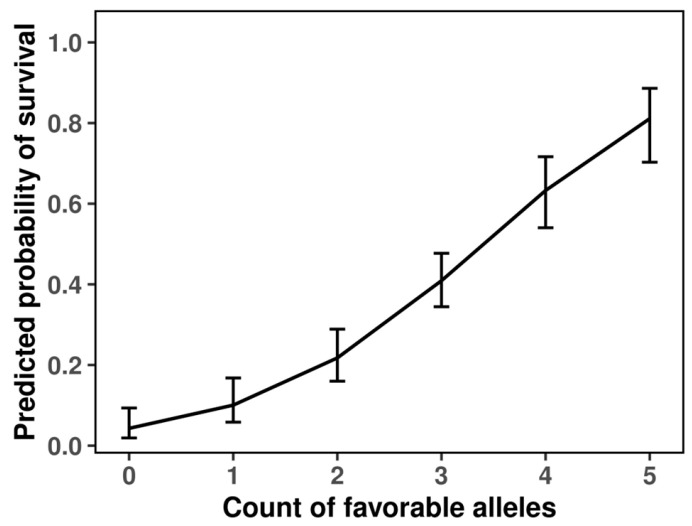
Additive effects of favorable alleles on predicted survival probability under frost stress.

When the four SNPs were combined into a cumulative genetic score, the probability of survival increased almost linearly with the total number of favorable alleles (Figure 4). Individuals carrying four or five favorable alleles had predicted survival probabilities above 0.7–0.8, while those lacking favorable alleles rarely exceeded 0.2. This clear dosage-dependent trend confirms the additive contribution of the loci and supports their joint role in determining survival outcomes.

These results are consistent with the statistical findings from the joint logistic regression model, which revealed significant additive effects without evidence of epistasis (*p* = 0.676). Thus, the identified loci not only show consistent directionality of effects but also collectively provide a reliable prediction of survival probability in the studied population.

**Figure 5:**
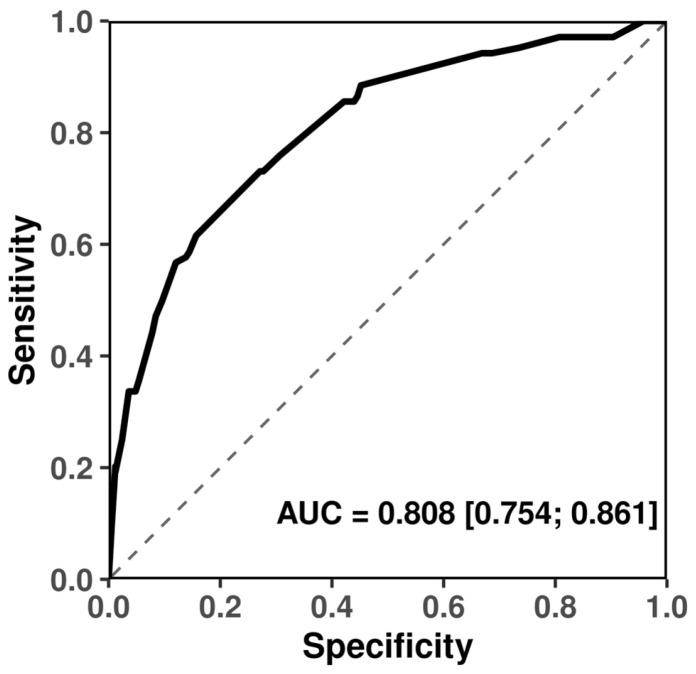
Receiver operating characteristic (ROC) curves comparing predictive performance of SNP-contained models predictors for frost tolerance in winter durum wheat.

The joint model containing all four SNPs exhibited a strong discriminative ability with AUC = 0.808 (95% CI: 0.754–0.861), whereas the baseline model without SNPs performed no better than chance (AUC = 0.500) (Figure 5). The difference between the two AUCs was highly significant (DeLong *p* = 1.53 × 10^−29^), confirming that inclusion of the four SNPs markedly improves predictive performance.

## 3. Discussion

Frost tolerance in winter durum wheat is a complex, multilayered trait. Despite the binary nature of the freezing-survival phenotype, the loci identified in this study demonstrated strong and stable signals across all post-GWAS analyses tailored to binary outcomes. Their significance, effect direction, and robustness were further reinforced by KASP validation for three loci, confirming that the associations reflect true underlying polymorphisms rather than artifacts of phenotypic resolution.

This study identified four genomic regions on chromosomes 1B, 5A, 5B, and 7B that collectively underpin frost tolerance in winter durum wheat. These loci represent interconnected layers of the cold stress response—encompassing signal perception, regulatory control, cellular protection, and translational maintenance. Rather than functioning independently, these processes likely operate as a coordinated network where the efficiency of one layer enhances or constrains another.

The variant *1B_41099587* lies in an intergenic region and was identified with a minor allele frequency (MAF) of 0.43, an effect size of 0.573, and phenotypic variance explained (PVE) of 9.4%. This SNP is in tight linkage with a cluster of genes encoding histidine-containing phosphotransfer (HPt) proteins. In *Arabidopsis thaliana* HPt proteins serve as intermediaries shuttling phosphoryl groups between sensor histidine kinases (e.g., AHKs) and response regulators (ARRs), thereby propagating environmental stress signals [17]. In the context of cold stress, such multistep two-component pathways are known to activate CBF transcription factors that govern cold-responsive genes [18].

The favorable variant A of *5B_517276534* is located in an intergenic region on chromosome 5B (MAF 0.28, effect size −0.861, PVE 7.6%), in proximity to genes encoding ornithine decarboxylase (ODC1B) and phospholipase D delta (PLDδ). Both enzymes are functionally linked to stress adaptation, especially under low temperatures [19,20]. Experimental studies in *Arabidopsis* have shown that altering PLDδ levels can significantly change frost tolerance: knockout of PLDδ renders plants more sensitive to frost, whereas overexpression of PLDδ enhances frost survival [21].

The SNP *7B_598228866* resides in an intergenic region adjacent to genes encoding the 40S ribosomal protein S14 (RPS14) and other ribosomal subunit proteins. It was detected at a lower frequency (MAF 0.09) but with a relatively large effect size (1.634) and PVE of 12.9%. Ribosomal proteins (RPs) have traditionally been viewed as structural components of the translation machinery; however, emerging evidence indicates that certain ribosomal subunits take on regulatory functions during stress adaptation [22].

The variant *5A_487200180* lies in the 5′ untranslated region of a gene encoding an E3 ubiquitin–protein ligase known as BIG BROTHER. E3 ubiquitin ligases are key regulators in plant stress response pathways, often mediating the turnover of critical signaling proteins [23]. It was identified at a MAF of 0.12 with a large effect size of 2.094 and phenotypic variance explained (PVE) of 21.5%. This locus had the highest effect size, underlining its biological significance, although direct KASP marker conversion proved challenging due to the tandem-repeat nature of the region. Such challenges are not uncommon in polyploid crops, where multi-copy genes and tandem repeats can hinder locus-specific amplification [24]. Notably, the *5A_487200180* locus maps approximately 30 Mb proximal to the canonical *Fr-A2* region (516–523 Mb) harboring the CBF gene cluster [11]. The E3 ubiquitin ligase BIG BROTHER identified at this position may modulate CBF pathway activity through protein degradation, as E3 ligases are known regulators of CBF stability and cold acclimation [25].

Evidence for such interdependence was provided by the detected strong, but non-significant interaction between the *5B_517276534* and *7B_598228866* loci (β = −0,833, *p* = 0.120). Recent studies indicate that the ODC pathway is closely linked to translational regulation, as polyamines derived from ODC activity stabilize ribosomes and enhance protein synthesis efficiency under stress conditions [26,27]. However, the relatively large magnitude of the interaction coefficient suggests the presence of a potential biological effect that may become evident with increased sample size.

Currently, only one genome-wide association study has investigated frost tolerance in winter durum wheat, namely the work of Sieber et al. (2016), which identified the *Fr-A2* and *Fr-B2* loci as the major determinants of frost tolerance [28]. Their results demonstrated that frost tolerance in winter durum is governed by the same molecular mechanisms as in bread wheat, particularly those centered on the CBF-dependent cold-responsive pathway and its regulatory connection to vernalization and photoperiod genes.

Given the limited number of studies on frost tolerance in winter durum wheat, findings from bread wheat, which exhibits highly similar genomic organization and cold-response pathways, can be considered representative for comparative analysis of corresponding loci. Comparative genomics between *Triticum* species provides a robust framework for transferring genomic information from one ploidy level to another, particularly between tetraploid durum wheat and hexaploid bread wheat. Due to their shared A-genome ancestry and the high degree of sequence conservation along homologous chromosomes, many trait-associated loci can be directly aligned between these species. Marcotuli et al. (2022) conducted a meta-analysis of GWAS in *Triticum turgidum* ssp. *durum*, identifying stable QTL hotspots and confirming strong collinearity between durum and bread wheat genomes through comparative mapping [29]. El Baidouri et al. (2017) further demonstrated that the A subgenome of *T. aestivum* is highly syntenic with the A genome of *T. turgidum*, reflecting their recent shared ancestry and structural conservation [30]. These findings justify direct positional comparison between loci on chromosome 5A of both species.

Concordance with *Fr*-regions is observed in the present study. The *5A_487200180* locus (E3 ubiquitin ligase BIG BROTHER) lies proximally to the major bread wheat frost-tolerance QTL reported by Soleimani et al. (2022) on 5A (*QTL_5A_2*, 516.45–523.45 Mb), which contains a dense cluster of CBF genes specifically mapping to the *Fr-2* region [31]. Similarly, Chen et al. (2019) located the similar QTL for winter survival on chromosome 5A in bread wheat (*QWs.ugw-5A.1*) [32]. Specifically, *QWs.ugw-5A.1* is positioned at about 499.66 Mb, only about 12 Mb away from *5A_487200180* locus. These loci likely belong to the same broader genomic interval on the long arm of chromosome 5A, which consistently harbors cold-responsive genes, including the CBF cluster at *Fr-2*.

On chromosome 5B, the *5B_517276534* locus (adjacent to ODC1B and PLDδ) is positioned ~25–30 Mb distal to the *Fr-B2* cluster (*QTL_5B*, 486.35–493.35 Mb) reported by Soleimani et al. (2022), which harbors multiple CBF homologs [31]. Genes involved in polyamine biosynthesis and phospholipid signaling may operate within the same regulatory framework as the CBF transcriptional network. Studies in tomato (*Solanum lycopersicum*) leaves have shown that these pathways exhibit coordinated but partly antagonistic expression patterns under cold stress, jointly contributing to membrane stabilization and cellular protection during frost conditions [33].

Although several plausible candidate genes were identified near the associated loci, mechanistic characterization of cold-response pathways requires transcriptomic or gene-editing approaches. The three KASP markers validated here directly support marker-assisted breeding for winter hardiness, while functional dissection of the underlying genes remains a promising direction for future research.

## 4. Materials and Methods

### 4.1. Plant Materials, Overall Design of Experiments and Phenotyping

All wheat cultivars were provided by the breeding program of the National Center of Grain named after P.P. Lukyanenko (Krasnodar, Russia). The segregating multi-family F_2_ panel of 270 wheat plants, representing a combined mixed population, was developed for genome-wide association analysis and validation of candidate loci associated with frost tolerance. The highly frost-tolerant cultivar Tsel and the frost-susceptible cultivar Senora participated as recurrent parental components. These cultivars were successively crossed with a diverse set of landraces (Odari, Andromeda, Zernograd, Leukurum, Pributkova, L2870VILLOSA, 4812h53, 4249h103, I-627494, 4598h48, 4291h83, 3902h3-18-3, I-627613, 3552h59-18-7, KN-21-130, 4754h37, 4743h81), forming a multi-parental structure that captured a broad range of frost-resistance and agronomic traits. Hybrid combinations included Tsel × landraces, Senora × landraces, and Tsel × Senora × landraces progenies, reflecting the diversity of germplasm. A total of plants were randomly selected from the segregating hybrid families for genotyping and phenotypic evaluation.

The evaluation of plants for frost tolerance was conducted using the direct frost method according to the Russian national standard for the evaluation of winter hardiness (Methodology for State Variety Trials of Agricultural Crops. Issue II: Cereal, Grain Legume, Maize, and Forage Crops, Moscow, 1989). The experiment was carried out in the C-816 frost chambers (Canada) installed in the phytotron complex of the National Center of Grain named after P.P. Lukyanenko (45°02′ N, 38°58′ E, Krasnodar, Russia).

**Figure 6:**
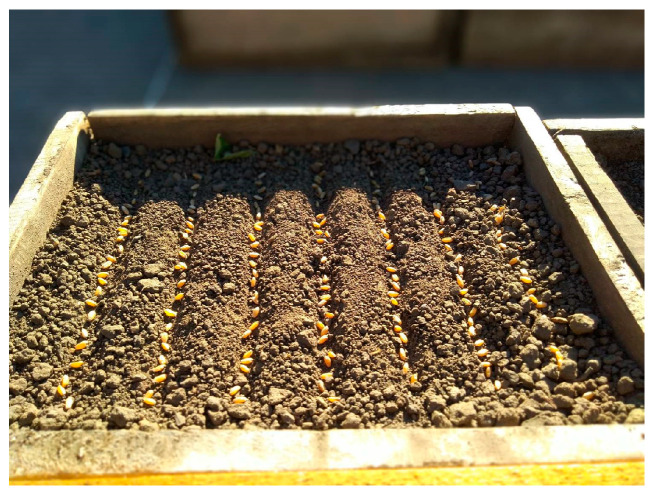
Sowing of winter durum wheat dry seeds in wooden boxes.

The individual plants intended for frost were grown in wooden boxes (38 × 26 × 12 cm) filled with a soil–sand mixture (3:1). Sowing was performed in October 2023, corresponding to the optimal regional sowing date, using dry seeds. Each box contained seven rows spaced 5 cm apart, with 25 seeds per row, sown to a depth of 2 cm (Figure 6). Emergence occurred 7 days later. The fourth row in each box was sown with the check variety Krupinka, known for its optimal frost tolerance among winter durum wheats (at a frost temperature of −15 °C, the average survival rate exceeds 50%) [34].

**Figure 7:**
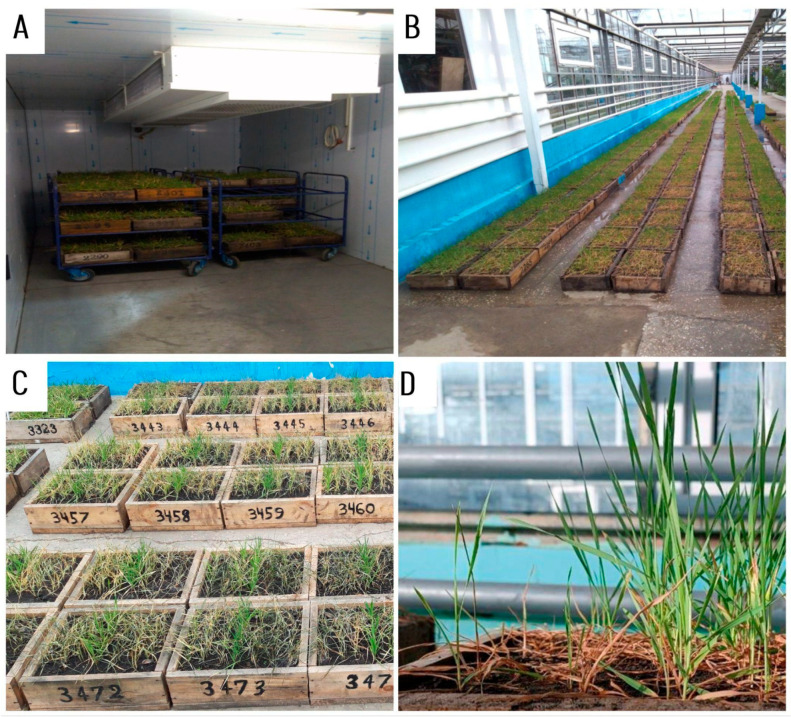
Experimental setup for frost tolerance evaluation in winter durum wheat. (**A**) Pre-hardening and frost in the C-816 programmable frost chamber. (**B**,**C**) Post-thaw recovery under phytotron conditions: (**B**) general view of recovery boxes and (**C**) layout of F_2_ plants used for survival scoring. (**D**) Close-up of regenerated tillers and newly emerged green leaves used as indicators of post-frost survival.

1. The entire experimental workflow consisted of three consecutive phases: outdoor acclimation, controlled freezing, and post-thaw recovery (Figure 7). Prior to freezing, plants underwent natural cold acclimation under field-like outdoor conditions during November and December. After acclimation, the freezing procedure was carried out in the C-816 programmable frost chamber. A 24 h pre-hardening stage at −2 to −5 °C preceded the main freezing cycle. The chamber temperature was then gradually lowered from −5 °C to −15 °C at a rate of approximately 1 °C per hour, after which the target semi-lethal temperature of −15 °C was maintained for 24 h to impose severe frost stress.
2. Following the freezing treatment, the temperature was increased stepwise from −15 °C to +5 °C over approximately two days to ensure gradual thawing and to avoid thermal shock. After thawing, plants were transferred to phytotron recovery conditions (20 °C day/16 °C night, 16 h photoperiod). Survival was recorded 7 and 14 days after freezing.

Finally, each individual plant within the F_2_ population was evaluated separately for survival following frost. Plants were scored as surviving (“1”) if they produced at least one new green leaf or tiller 7–14 days post-thaw; otherwise, they were scored as dead (“0”). Among the 270 individuals, 104 plants survived (38.5%) and 166 did not survive (61.5%). These binary survival data were subsequently used for association analysis to identify loci linked to frost tolerance. Among the survivors, most individuals were derived from Tsel (66.3%), while 21.2% originated from Senora and 12.5% had mixed ancestry (Tsel + Senora). Among the non-surviving plants, 52.4% were of Tsel origin, 34.3% from Senora, and 13.3% had mixed ancestry.

### 4.2. Genotyping and SNP Calling

Genomic DNA was extracted from dried leaf tissue using the “MagnoPrime GMO” kit following the manufacturer’s protocol. GBS libraries were prepared following the protocol described by Elshire et al. (2011), using the restriction enzymes MspI and PstI, and sequenced on an Illumina NovaSeq 6000 platform [35]. Raw reads were aligned to the durum wheat reference genome (Svevo v2.0 [36]) using bowtie 2 v2.3.5 with standard parameters [37].

Variant calling was performed using ChoCallate (https://github.com/alermol/ChoCallate; accessed 6 June 2025), an automated, high-performance ensemble framework developed by our group for consensus-based variant discovery, integrating multiple variant callers to generate robust, high-confidence SNVs and INDELs. The raw VCF was processed using PLINK v2.00a6LM [38]. Only biallelic variants were retained, resulting in 278,727 SNPs. Quality control was applied in several stages: variants with a missing rate above 20% were excluded, leaving 70,796 variants. Variants with minor allele frequency (MAF) < 0.05 were filtered out, yielding 12.108 sites, and loci with heterozygosity > 30% were removed, leaving 9,529 variants. Missing genotypes were imputed using Beagle, followed by a secondary MAF ≥ 0.05 filter, resulting in 9.446 high-quality polymorphic variants (8,388 SNPs and 1,058 indels) used for GWAS.

### 4.3. Genome-Wide Association Analysis

Genome-wide association analysis (GWAS) was performed using the GAPIT v3 package [39]. The Bayesian-information and Linkage-disequilibrium Iteratively Nested Keyway (BLINK) model was applied to identify significant associations between SNP markers and the frost tolerance phenotype [40]. To account for population structure, the first five principal components (PCs) derived from the genotype data were included as covariates in the model.

Significance thresholds were determined using a false discovery rate (FDR) 10% threshold to control for multiple testing. Manhattan and quantile–quantile (Q-Q) plots were generated using GAPIT’s visualization functions. Effect sizes, standard errors, and the percentage of phenotypic variance explained (PVE) were extracted for all significant SNPs. Candidate genes were identified based on the physical position of significant SNPs in the Svevo v2.0. SNP annotation was performed using SnpEff with default parameters [41].

### 4.4. Designing KASP Markers

Specific KASP markers were developed for the significant SNPs identified in the GWAS to validate the detected associations. Primer sets were designed according to LGC Genomics guidelines, flanking sequences retrieved from the *Triticum durum* Svevo v2.0 reference genome. Each assay included two allele-specific forward primers containing the universal FAM or HEX tails at the 5′ end, and a single common reverse primer. All KASP reactions were carried out in 5 μL volume using CFX96 Real-Time PCR System ( Bio-Rad, Hercules, CA, USA) under the following cycling conditions: 94 °C for 15 min, followed by 10 touchdown cycles (94 °C for 20 s; annealing starting at 61 °C and decreasing by 0.6 °C per cycle), and 26 cycles at 94 °C for 20 s, 55 °C for 60 s. Fluorescence was measured at the end of each annealing step, and endpoint genotyping clusters were analyzed using Bio-Rad CFX Manager software (v3.1).

### 4.5. Post-GWAS Analysis

Because each F_2_ plant represents a unique genotype subjected to a destructive freezing event, frost tolerance could only be recorded as a binary survival response. To ensure that this limited phenotypic resolution did not bias inference, we applied a series of complementary statistical procedures beyond the primary GWAS, including logistic regression, likelihood-ratio testing, comparison of alternative inheritance models, and assessment of potential epistasis. In addition, significant SNPs were converted to KASP markers to provide an independent validation layer and confirm the robustness of genotype–phenotype associations.

Post-GWAS validation was conducted to evaluate the contribution of individual SNPs to survival probability and to assess the combined predictive power of significant loci. All analyses were performed in R (v4.2.2) using the packages stats, broom (v1.0.6), dplyr (v1.1.4), tidyr (v1.3.1), ggplot2 (v3.5.1), and pROC (v1.18.5).

Binary survival outcomes were modeled using logistic regression (GLM with binomial link). For each SNP, the genotype was coded as the minor-allele dosage (0, 1, 2). The full multivariate model included all significant loci identified in the GWAS.

#### 4.5.1. Inheritance Model Comparison

For each SNP, three alternative genetic models were fitted: additive (dosage-related), dominant (carrier vs. non-carrier), and genotypic (3-level categorical). Each model was compared against the intercept-only model using LRTs, and pseudo-R^2^ values were calculated to assess the best-fitting mode of inheritance.

#### 4.5.2. Integrated Evaluation of Joint and Locus-Specific Effects

The predictive value of the SNP set was first assessed by contrasting the full additive logistic model (including all SNP terms) with an intercept-only model using a global likelihood-ratio test. Model improvement was summarized by McFadden’s pseudo-R^2^ [42].

Locus-specific contributions within the multivariable framework were then quantified by type-II likelihood-ratio tests (LRTs) [43], and for each SNP a partial pseudo-R^2^ was obtained by refitting the model after removing the focal term. Effect sizes were reported as logit coefficients (β) with standard errors (SEs), Wald *p*-values, odds ratios (OR = eβ), and 95% confidence intervals derived via broom package. Multiplicity for per-marker tests was controlled using the Benjamini–Hochberg false discovery rate.

To test for non-additive (pairwise) interactions between loci, an extended model including all two-way SNP × SNP terms was fitted and compared to the additive model via LRT. The change in model deviance and pseudo-R^2^ was used to quantify epistatic effects.

#### 4.5.3. Model Discrimination

Discriminatory performance was assessed using receiver operating characteristic (ROC) analysis implemented in the pROC package [44]. The area under the ROC curve (AUC) and its 95% confidence interval were calculated using DeLong’s method [45].

### 4.6. Generative AI Statement

No generative artificial intelligence (GenAI) was used in the creation of this manuscript.

## 5. Conclusions

This study uncovered four genomic loci on chromosomes 1B, 5A, 5B, and 7B that collectively underpin frost tolerance in winter durum wheat. We demonstrated that *1B_41099587* (HPt signaling), *5B_517276534* (linked to phospholipid and polyamine metabolism), *5A_487200180* (E3 ubiquitin ligase BIG BROTHER), and *7B_598228866* (linked to ribosomal regulation) loci consistently explained a substantial portion of phenotypic variation in winter survival (ΔMcFadden R^2^ = 0.230). Three of these (*1B_41099587*, *5B_517276534*, *7B_598228866*) were converted into KASP markers. The additive mode of inheritance and the absence of pronounced epistasis indicate that favorable alleles can be pyramidized through conventional breeding or genomic selection to achieve cumulative improvement in frost tolerance. The validated markers provide direct applicability for marker-assisted selection in durum wheat breeding programs targeting continental climates.

## Figures and Tables

**Table 1:** Significant SNPs associated with frost tolerance in winter durum wheat.

Chromosome	Position	REF	ALT	*p*-Value	MAF	PVE, %
1B	41,099,587	T	C	5.79 × 10^−7^	0.431	9.388
5A	487,200,180	G	T	8.21 × 10^−12^	0.124	21.517
5B	517,276,534	A	G	3.80 × 10^−6^	0.276	7.568
7B	598,228,866	C	A	6.25 × 10^−6^	0.098	12.937

**Table 2:** The KASP primers for detecting SNP alleles.

SNP	Primer	Sequence (5′->3′)	Tm (°C)	GC (%)
1B_41099587	A	CCGTCCATGTTCTCTCGCAT	60	55
	B	CGTCCATGTTCTCTCGCAC	60	58
	C	CTGGATCCATCACGCCAAG	60	58
5B_517276534	A	GGATGAGGATGAGCAAAGCAA	60	48
	B	GGATGAGGATGAGCAAAGCAG	61	52
	C	CCATGCATGGGCCACTTTTTC	61	52
7B_598228866	A	GGTCCGAATGTTATAAGGCCC	61	52
	B	GGTCCGAATGTTATAAGGCCA	60	48
	C	CGAAATCGACCTACAAACTGTTG	61	43

**Table 3:** Likelihood ratio test comparing the additive 4-SNP model with the intercept-only model for frost tolerance in winter durum wheat.

Comparison	LRT χ^2^	df	*p*-Value	ΔMcFadden R^2^
4-SNP model vs. Intercept-only	82.7	4	4.76 × 10^−17^	0.230

**Table 4:** Logistic regression statistics and effect estimates for SNPs significantly associated with frost tolerance in winter durum wheat.

SNP	LRT χ^2^	df	*p*-Value	Effect (β)	ΔMcFadden R^2^	OR
5B_517276534	17.92	1	3.07 × 10^−5^	−0.861	0.050	0.42
7B_598228866	20.11	1	1.46 × 10^−5^	1.633	0.056	5.12
1B_41099587	10.48	1	1.20 × 10^−3^	0.572	0.029	1.77
5A_487200180	42.32	1	3.09 × 10^−10^	2.094	0.118	8.11

**Table 5:** Likelihood ratio test comparing the additive 4-SNP model with the additive plus all two-way interaction 4-SNP model for frost tolerance.

Comparison	df	*p*-Value	ΔMcFadden R^2^
Additive + All 2-way Interactions	6	0.676	0.011

**Table 6:** Logistic regression coefficients for the interaction model 7B_598228866 × 5B_517276534.

Term	Effect (β)	Std. Error	Pr(>|z|)
(Intercept)	−0.401	0.171	0.019
7B_598228866	1.624	0.446	0.000
5B_517276534	−0.615	0.213	0.004
7B_598228866:5B_517276534	−0.833	0.536	0.120

## Data Availability

The data supporting the findings of this study can be found in Table S1 and Data S1. Table S1 provides a comparison of inheritance models (additive, dominant, and genotypic) for SNPs associated with frost tolerance in durum wheat. Data S1 contains a VCF file with four representative SNPs extracted from the original variant dataset. No additional publicly archived datasets were generated or analyzed during this study.

## Supplementary Materials

The following supporting information can be downloaded at: https://www.mdpi.com/article/10.3390/plants15010019/s1, Data S1: VCF file containing four representative SNPs extracted from the original dataset. Table S1: Model comparison of inheritance patterns (additive, dominant, and genotypic) for SNPs associated with frost tolerance in durum wheat.

## Abbreviations

The following abbreviations are used in this manuscript:

AHK	Arabidopsis Histidine Kinase
ARR	Arabidopsis Response Regulator
AUC	Area Under the ROC Curve
BLINK	Bayesian-information and Linkage-disequilibrium Iteratively Nested Keyway
CBF	C-repeat Binding Factor
CI	Confidence Interval
DF	Degrees of Freedom
FDR	False Discovery Rate
Fr-1	Frost Resistance locus 1
Fr-2	Frost Resistance locus 2
Fr-A2	Frost Resistance locus A2 (on 5A)
Fr-B2	Frost Resistance locus B2 (on 5B)
GBS	Genotyping-by-Sequencing
GLM	Generalized Linear Model
GWAS	Genome-Wide Association Study
HOS1	High Expression of Osmotically Responsive Genes 1
HPt	Histidine-containing Phosphotransfer protein
ICE1	Inducer of CBF Expression 1
INDEL	Insertion/Deletion
KASP	Kompetitive Allele-Specific PCR
LD	Linkage Disequilibrium
LRT	Likelihood Ratio Test
MAF	Minor Allele Frequency
MAS	Marker-Assisted Selection
Mb	Megabase
NTC	No Template Control
ODC	Ornithine Decarboxylase
ODC1B	Ornithine Decarboxylase 1B
OR	Odds Ratio
PCA	Principal Component Analysis
PCR	Polymerase Chain Reaction
PLDδ	Phospholipase D delta
Ppd-A1	Photoperiod gene A1
Ppd-B1	Photoperiod gene B1
PVE	Phenotypic Variance Explained
Q-Q plot	Quantile–Quantile plot
QTL	Quantitative Trait Locus
RFU	Relative Fluorescence Units
RPS14	Ribosomal Protein S14
SE	Standard Error
SNP	Single-Nucleotide Polymorphism
SNV	Single-Nucleotide Variant
UTR	Untranslated Region
VCF	Variant Call Format
Vrn-A1	Vernalization gene A1

## References

[1] Peters Haugrud A.R., Achilli A.L., Martínez-Peña R., Klymiuk V. (2025). Future of Durum Wheat Research and Breeding: Insights from Early Career Researchers. Plant Genome.

[2] Martínez-Moreno F., Ammar K., Solís I. (2022). Global Changes in Cultivated Area and Breeding Activities of Durum Wheat from 1800 to Date: A Historical Review. Agronomy.

[3] Longin C.F.H., Sieber A.-N., Reif J.C. (2013). Combining Frost Tolerance, High Grain Yield and Good Pasta Quality in Durum Wheat. Plant Breed..

[4] Kroupina A.Y., Yanovsky A.S., Korobkova V.A., Bespalova L.A., Arkhipov A.V., Bukreeva G.I., Voropaeva A.D., Kroupin P.Y., Litvinov D.Y., Mudrova A.A. (2023). Allelic Variation of Glu-A1 and Glu-B1 Genes in Winter Durum Wheat and Its Effect on Quality Parameters. Foods.

[5] Richetti J., Sadras V.O., He D., Leske B., Hu P., Beletse Y., Cossani C.M., Nguyen H., Zheng B., Deery D.M. (2025). Challenges in Modelling the Impact of Frost and Heat Stress on the Yield of Cool-Season Annual Grain Crops. Front. Plant Sci..

[6] Cann D.J., Schillinger W.F., Hunt J.R., Porker K.D., Harris F.A.J. (2020). Agroecological Advantages of Early-Sown Winter Wheat in Semi-Arid Environments: A Comparative Case Study from Southern Australia and Pacific Northwest United States. Front. Plant Sci..

[7] Slafer G.A., Savin R., Sadras V.O. (2023). Wheat Yield Is Not Causally Related to the Duration of the Growing Season. Eur. J. Agron..

[8] Beres B.L., Rahmani E., Clarke J.M., Grassini P., Pozniak C.J., Geddes C.M., Porker K.D., May W.E., Ransom J.K. (2020). A Systematic Review of Durum Wheat: Enhancing Production Systems by Exploring Genotype, Environment, and Management (G × E × M) Synergies. Front. Plant Sci..

[9] Grosse-Heilmann M., Cristiano E., Deidda R., Viola F. (2024). Durum Wheat Productivity Today and Tomorrow: A Review of Influencing Factors and Climate Change Effects. Resour. Environ. Sustain..

[10] Hassan M.A., Xiang C., Farooq M., Muhammad N., Yan Z., Hui X., Yuanyuan K., Bruno A.K., Lele Z., Jincai L. (2021). Cold Stress in Wheat: Plant Acclimation Responses and Management Strategies. Front. Plant Sci..

[11] Würschum T., Longin C.F.H., Hahn V., Tucker M.R., Leiser W.L. (2017). Copy Number Variations of CBF Genes at the Fr-A2 Locus Are Essential Components of Winter Hardiness in Wheat. Plant J..

[12] Gupta P., Kabbaj H., El Hassouni K., Maccaferri M., Sanchez-Garcia M., Tuberosa R., Bassi F.M. (2020). Genomic Regions Associated with the Control of Flowering Time in Durum Wheat. Plants.

[13] Ferrante A., Cullis B.R., Smith A.B., Able J.A. (2021). A Multi-Environment Trial Analysis of Frost Susceptibility in Wheat and Barley Under Australian Frost-Prone Field Conditions. Front. Plant Sci..

[14] Babben S., Schliephake E., Janitza P., Berner T., Keilwagen J., Koch M., Arana-Ceballos F.A., Templer S.E., Chesnokov Y., Pshenichnikova T. (2018). Association Genetics Studies on Frost Tolerance in Wheat (*Triticum aestivum* L.) Reveal New Highly Conserved Amino Acid Substitutions in CBF-A3, CBF-A15, VRN3 and PPD1 Genes. BMC Genom..

[15] Ermolaev A., Bespalova L., Korobkova V., Yanovsky A., Nazarova L., Kroupina A., Chernook A., Mudrova A., Voronezhskaya V., Kroupin P. (2025). High-Quality Bonds: Serine Acetyltransferase 2 Gene Revealed by GWAS Is Associated with Grain Protein Content in Spring Durum Wheat. Front. Plant Sci..

[16] Dias-Fields L., Adamala K.P. (2022). Engineering Ribosomes to Alleviate Abiotic Stress in Plants: A Perspective. Plants.

[17] Suzuki T., Imamura A., Ueguchi C., Mizuno T. (1998). Histidine-Containing Phosphotransfer (HPt) Signal Transducers Implicated in His-to-Asp Phosphorelay in Arabidopsis. Plant Cell Physiol..

[18] Feng Y., Li Z., Kong X., Khan A., Ullah N., Zhang X. (2025). Plant Coping with Cold Stress: Molecular and Physiological Adaptive Mechanisms with Future Perspectives. Cells.

[19] Vergnolle C., Vaultier M.-N., Taconnat L., Renou J.-P., Kader J.-C., Zachowski A., Ruelland E. (2005). The Cold-Induced Early Activation of Phospholipase C and D Pathways Determines the Response of Two Distinct Clusters of Genes in Arabidopsis Cell Suspensions. Plant Physiol..

[20] Amini S., Maali-Amiri R., Kazemi-Shahandashti S.-S., López-Gómez M., Sadeghzadeh B., Sobhani-Najafabadi A., Kariman K. (2021). Effect of Cold Stress on Polyamine Metabolism and Antioxidant Responses in Chickpea. J. Plant Physiol..

[21] Li W., Li M., Zhang W., Welti R., Wang X. (2004). The Plasma Membrane-Bound Phospholipase Ddelta Enhances Freezing Tolerance in Arabidopsis Thaliana. Nat. Biotechnol..

[22] Stępiński D. (2025). Decoding Plant Ribosomal Proteins: Multitasking Players in Cellular Games. Cells.

[23] Wang S., Lv X., Zhang J., Chen D., Chen S., Fan G., Ma C., Wang Y. (2022). Roles of E3 Ubiquitin Ligases in Plant Responses to Abiotic Stresses. Int. J. Mol. Sci..

[24] Makhoul M., Rambla C., Voss-Fels K.P., Hickey L.T., Snowdon R.J., Obermeier C. (2020). Overcoming Polyploidy Pitfalls: A User Guide for Effective SNP Conversion into KASP Markers in Wheat. Theor. Appl. Genet..

[25] Dong C.-H., Agarwal M., Zhang Y., Xie Q., Zhu J.-K. (2006). The Negative Regulator of Plant Cold Responses, HOS1, is a RING E3 Ligase That Mediates the Ubiquitination and Degradation of ICE1. Proc. Natl. Acad. Sci. USA.

[26] Igarashi K., Ito K., Sakai Y., Ogasawara T., Kashiwagi K., Zappia V., Pegg A.E. (1988). Regulation of Protein Synthesis by Polyamines. Progress in Polyamine Research.

[27] Li C.H., Ohn T., Ivanov P., Tisdale S., Anderson P. (2010). eIF5A Promotes Translation Elongation, Polysome Disassembly and Stress Granule Assembly. PLoS ONE.

[28] Sieber A.-N., Longin C.F.H., Leiser W.L., Würschum T. (2016). Copy Number Variation of CBF-A14 at the Fr-A2 Locus Determines Frost Tolerance in Winter Durum Wheat. Theor. Appl. Genet..

[29] Marcotuli I., Soriano J.M., Gadaleta A. (2022). A Consensus Map for Quality Traits in Durum Wheat Based on Genome-Wide Association Studies and Detection of Ortho-Meta QTL across Cereal Species. Front. Genet..

[30] El Baidouri M., Murat F., Veyssiere M., Molinier M., Flores R., Burlot L., Alaux M., Quesneville H., Pont C., Salse J. (2017). Reconciling the Evolutionary Origin of Bread Wheat (*Triticum aestivum*). New Phytol..

[31] Soleimani B., Lehnert H., Babben S., Keilwagen J., Koch M., Arana-Ceballos F.A., Chesnokov Y., Pshenichnikova T., Schondelmaier J., Ordon F. (2022). Genome Wide Association Study of Frost Tolerance in Wheat. Sci. Rep..

[32] Chen Y., Sidhu H.S., Kaviani M., McElroy M.S., Pozniak C.J., Navabi A. (2019). Application of Image-Based Phenotyping Tools to Identify QTL for in-Field Winter Survival of Winter Wheat (*Triticum aestivum* L.). Theor. Appl. Genet..

[33] Upadhyay R.K., Fatima T., Handa A.K., Mattoo A.K. (2020). Polyamines and Their Biosynthesis/Catabolism Genes Are Differentially Modulated in Response to Heat Versus Cold Stress in Tomato Leaves (*Solanum lycopersicum* L.). Cells.

[34] Ivanisova A., Marchenko D., Kostylenko O., Dubinina O., Antonenko L. (2023). Studying Varieties of Winter Durum Wheat in Interstation Test on Economic and Valuable Features. E3S Web Conf..

[35] Elshire R.J., Glaubitz J.C., Sun Q., Poland J.A., Kawamoto K., Buckler E.S., Mitchell S.E. (2011). A Robust, Simple Genotyping-by-Sequencing (GBS) Approach for High Diversity Species. PLoS ONE.

[36] Maccaferri M., Harris N.S., Twardziok S.O., Pasam R.K., Gundlach H., Spannagl M., Ormanbekova D., Lux T., Prade V.M., Milner S.G. (2019). Durum Wheat Genome Highlights Past Domestication Signatures and Future Improvement Targets. Nat. Genet..

[37] Langmead B., Salzberg S.L. (2012). Fast Gapped-Read Alignment with Bowtie 2. Nat. Methods.

[38] Purcell S., Neale B., Todd-Brown K., Thomas L., Ferreira M.A.R., Bender D., Maller J., Sklar P., de Bakker P.I.W., Daly M.J. (2007). PLINK: A Tool Set for Whole-Genome Association and Population-Based Linkage Analyses. Am. J. Hum. Genet..

[39] Wang J., Zhang Z. (2021). GAPIT Version 3: Boosting Power and Accuracy for Genomic Association and Prediction. Genom. Proteom. Bioinform..

[40] Huang M., Liu X., Zhou Y., Summers R.M., Zhang Z. (2019). BLINK: A Package for the next Level of Genome-Wide Association Studies with Both Individuals and Markers in the Millions. GigaScience.

[41] Cingolani P., Platts A., Wang L.L., Coon M., Nguyen T., Wang L., Land S.J., Lu X., Ruden D.M. (2012). A Program for Annotating and Predicting the Effects of Single Nucleotide Polymorphisms, SnpEff. Fly.

[42] Hughes G., Choudhury R.A., McRoberts N. (2019). Summary Measures of Predictive Power Associated with Logistic Regression Models of Disease Risk. Phytopathology.

[43] Qian M., Shao Y. (2013). A Likelihood Ratio Test for Genomewide Association under Genetic Heterogeneity. Ann. Hum. Genet..

[44] Nahm F.S. (2022). Receiver Operating Characteristic Curve: Overview and Practical Use for Clinicians. Korean J. Anesth..

[45] Zhu H., Liu S., Xu W., Dai J., Benbouzid M. (2024). Linearithmic and Unbiased Implementation of DeLong’s Algorithm for Comparing the Areas under Correlated ROC Curves. Expert Syst. Appl..

