# Supplementary material for: Genetic Dissection of Frost Tolerance in Winter Durum Wheat: Three Validated KASP Markers for Marker-Assisted Selection
Source: Plants (Basel). 2025 Dec 20;15(1):19. doi: 10.3390/plants15010019 (PMC12787326; doi:10.3390/plants15010019)
Supplement: Supplementary file 1 [file plants-15-00019-s001.zip › Supplementary_Table_S1.pdf]

Supplementary Table 1. Model comparison of inheritance patterns (additive, dominant, and genotypic) for SNPs associated with frost tolerance in durum wheat.

Marker	Model	df	ΔMcFadden R^2	P-value
1B_41099587	additive	1	0.0147	2.16×10^{-2}
	dominant	1	0.0143	2.33×10^{-2}
	genotypic	2	0.0154	6.26×10^{-2}
7B_598228866	additive	1	0.0361	3.15×10^{-4}
	dominant	1	0.0361	3.15×10^{-4}
	genotypic	2	0.0361	3.15×10^{-4}
5B_517276534	additive	1	0.0470	3.88×10^{-5}
	dominant	1	0.0355	3.54×10^{-4}
	genotypic	2	0.0482	1.71×10^{-4}
5A_487200180	additive	1	0.110	3.18×10^{-10}
	dominant	1	0.108	4.23×10^{-10}
	genotypic	2	0.110	2.41×10^{-9}
